# Proteomic assessment of a cell model of spinal muscular atrophy

**DOI:** 10.1186/1471-2202-12-25

**Published:** 2011-03-08

**Authors:** Chia-Yen Wu, Dosh Whye, Lisa Glazewski, Leila Choe, Douglas Kerr, Kelvin H Lee, Robert W Mason, Wenlan Wang

**Affiliations:** 1 Department of Biological Science, University of Delaware, Newark, DE, USA; 2 Department of Pediatrics, Columbia University Medical Center, New York, NY, USA; 3 Nemours Biomedical Research, Nemours/Alfred I. duPont Hospital for Children, Wilmington, DE, USA; 4 Delaware Biotechnology Institute, Newark, DE, USA; 5 Experimental Neurology, Biogen Idec, Cambridge, MA, USA; 6 Department of Pediatrics, Thomas Jefferson University, Philadelphia, PA, USA

## Abstract

**Background:**

Deletion or mutation(s) of the survival motor neuron 1 *(SMN1) *gene causes spinal muscular atrophy (SMA), a neuromuscular disease characterized by spinal motor neuron death and muscle paralysis. Complete loss of the SMN protein is embryonically lethal, yet reduced levels of this protein result in selective death of motor neurons. Why motor neurons are specifically targeted by SMN deficiency remains to be determined. In this study, embryonic stem (ES) cells derived from a severe SMA mouse model were differentiated into motor neurons *in vitro *by addition of retinoic acid and sonic hedgehog agonist. Proteomic and western blot analyses were used to probe protein expression alterations in this cell-culture model of SMA that could be relevant to the disease.

**Results:**

When ES cells were primed with Noggin/fibroblast growth factors (bFGF and FGF-8) in a more robust neural differentiation medium for 2 days before differentiation induction, the efficiency of *in vitro *motor neuron differentiation was improved from ~25% to ~50%. The differentiated ES cells expressed a pan-neuronal marker (neurofilament) and motor neuron markers (Hb9, Islet-1, and ChAT). Even though SMN-deficient ES cells had marked reduced levels of SMN (~20% of that in control ES cells), the morphology and differentiation efficiency for these cells are comparable to those for control samples. However, proteomics in conjunction with western blot analyses revealed 6 down-regulated and 14 up-regulated proteins with most of them involved in energy metabolism, cell stress-response, protein degradation, and cytoskeleton stability. Some of these activated cellular pathways showed specificity for either undifferentiated or differentiated cells. Increased p21 protein expression indicated that SMA ES cells were responding to cellular stress. Up-regulation of p21 was confirmed in spinal cord tissues from the same SMA mouse model from which the ES cells were derived.

**Conclusion:**

SMN-deficient ES cells provide a cell-culture model for SMA. SMN deficiency activates cellular stress pathways, causing a dysregulation of energy metabolism, protein degradation, and cytoskeleton stability.

## Background

Spinal muscular atrophy (SMA) is an autosomal recessive disorder with a prevalence of 1 in 6000 live births and a carrier incidence of 1 in 40-50 [1,2]. The hallmark of SMA is death of spinal motor neurons and progressive muscle atrophy [3]. Based on age of onset and clinical severity, childhood SMA has been classified into types I, II, and III [2,4]. Type I SMA is the most severe, resulting in the death of the child before the age of two, while type II and III individuals can live on into adulthood; however, they suffer from varying degrees of muscle paralysis and atrophy.

Genetic analyses of familial SMA indicate that the vast majority of SMA is caused by deletion or mutation(s) of the telomeric copy of the survival motor neuron 1 (*SMN1*) gene [5]. Complete loss of this gene in all species is lethal. In humans, a highly homologous centromeric copy of the *SMN *gene, *SMN2*, enables patient survival, but it cannot completely compensate for the loss of *SMN1 *[6,7]. The encoded SMN protein has been shown to play an essential role in the assembly of small nuclear ribonucleoprotein (snRNP) complexes [8-10]. SMN appears to function in snRNP biogenesis by interacting with Gemins 2-8, and Unrip [8,11,12]. A correlation between snRNP assembly activity in the spinal cord of SMA mice and severity of the disease has been demonstrated [13]. Widespread pre-mRNA splicing defects have also been seen in many cells and tissues in an SMA mouse model, indicating that SMA may be a general splicing disorder [14].

Reduced levels of SMN in SMA patients and animal models result in selective death of motor neurons, indicating that SMN plays a more critical role in motor neurons. Consistent with this indication, SMN has been shown to localize to granules that are actively transported into neurites and growth cones [15]. Axonal SMN appears to associate with heterogenous nuclear ribonucleoprotein R (hnRNP R) and to be involved in the transport of β-actin mRNA [16]. Indeed, distal axons and growth cones of motor neurons from SMA mice have defects in neurite outgrowth and reduced levels of β-actin mRNA and protein [16]. Zebra fish motor neurons with SMN deficiency also exhibit shorter and/or abnormally branched axons [17,18]. Recent studies in SMA mouse models further identified pre-synaptic defects including poor arborization, intermediate filament aggregation, impaired synaptic vesicle release, and trunk denervation [19-24]. Collectively, these data support a specific function for SMN in motor neurons and neuromuscular junctions.

Several SMA mouse models have been developed in the past decade [24-27]. One severe SMA mouse model (*SMN2*^+/+^*Smn*^-/-^) most closely mimics human type I SMA in that it lacks the mouse *Smn *gene but carries two copies of the human *SMN2 *gene [25]. The SMA pups with this genotype appear normal at birth, but at post-natal day 2 (P2), they develop SMA-like symptoms including reduced suckling, decreased movement, and labored breathing. The pups die by P6-7. The short lifespan in this SMA mouse prohibits wide use of this model for mechanistic studies or drug development for SMA. Thus, development of an *in vitro *cell-culture system from this transgenic mouse, that recapitulates motor neuron differentiation and the unique features of mature motor neurons such as extension of axons and formation of neuromuscular junctions, will enable us to study motor neuron-specific functions of SMN.

Embryonic stem (ES) cells are unique cells derived from the inner cell mass of the mammalian blastocyst. These cells are pluripotent, immortal, and can be differentiated into mature cell types in response to specific cues [28,29]. Generation of spinal neurons can be achieved by exposure of embryoid bodies (EBs) to retinoic acid (RA) and sonic hedgehog (Shh) or a hedgehog agonist [30,31]. Motor neurons differentiated via this protocol were found to extend axons and form synapses with target muscle when implanted in the spinal cord [31-34], thus confirming the power of this approach in generating mature neuronal subpopulations.

In this study, we report on the effects of the differentiation of ES cells derived from the severe SMA mouse model into motor neurons. We developed a new protocol to improve differentiation efficiency from ~25% to ~50%. The differentiated motor neurons were characterized by immunofluorescence and western blotting of neuronal and motor neuron markers. Using proteomic techniques, we determined that multiple signaling pathways are dysregulated in SMA cells.

## Results

### *In vitro *motor neuron differentiation is enhanced by increasing neural patterning with Noggin and fibroblast growth factors

**Figure 1:**
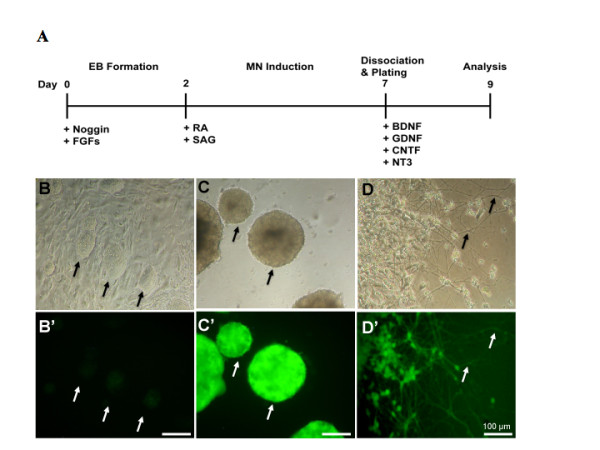
**Differentiation of murine ES cells into motor neurons**. The scheme outlines the process of motor neuron differentiation from ES cells (A). Murine ES cells (HBG3) were expanded on a layer of primary mouse embryonic fibroblasts. Undifferentiated stem cells form round clusters (arrows) as they proliferate on top of a feeding fibroblast layer, and they have weak GFP expression (B-B'). Stem cells were separated from the fibroblast layer and plated in low attachment dishes to allow for differentiation. Two-day-old stem cell aggregates (called embryoid bodies, EB, arrows) were induced to differentiate with 1 μM retinoic acid and 1 μM sonic hedgehog agonist SAG for 5 days. Differentiated ES cells have strong GFP expression (C-C'). When plated on poly-DL-ornithine/laminin/matrigel coated plates, the differentiated ES cells extend long neurites after 2 days in culture (D-D'). Representative phase contrast images for 1B-1D are shown on the top and corresponding fluorescent images are on the bottom. Scale bar = 200 μm except as indicated. MN = motor neuron.

To establish a cell-culture model of SMA, we initially used the control ES line (HBG3; HB9::eGFP) to optimize *in vitro *conditions for differentiation of ES cells into motor neurons. Since HBG3 ES cells were derived from a transgenic mouse expressing eGFP under the control of motor neuron specific promoter Hb9 [31], the efficiency of differentiation can be monitored by measuring the population of GFP^+ ^cells. Initially, ES cells were propagated on mouse embryonic fibroblasts and differentiated by the addition of RA and a Shh agonist (smoothened agonist, SAG) using a previous published protocol [31]. Retinoic acid caudalizes neural progenitors, while Shh has a ventralizing effect, leading to differentiation into motor neurons [31]. We optimized concentrations of RA (0.001-2 μM) and SAG (0.001-1 μM) and found that a combination of 1 μM RA and 1 μM SAG gave the best degree of differentiation (~25%). Purmorphamine, a compound that activates the hedgehog signaling pathway and directs motor neuron differentiation [35,36], was also tested at 1-2.5 μM concentrations in combination with 1 μM RA, but the differentiation rate was always less than 20%. In order to enhance the neuralization process, we primed the ES cells with the bone morphogenetic protein antagonist, Noggin, and with bFGF and FGF-8 (see Methods) prior to the 5-day induction period with RA and SAG (Figure 1A). Noggin is implicated in neural induction according to the default model of neurogenesis and results in the formation of anterior neural patterning, while FGF acts synergistically with Noggin in inducing neural tissue formation by promoting a posterior neural identity [37-40]. Priming ES cells with these two signaling molecules before differentiation induction significantly increased GFP^+ ^population from 24.8% ± 8.1 to 51% ± 0.8% (n = 3; *p *< 0.01, Student's *t-*test). The differentiated ES cells extend long processes when plated on poly-ornithine/laminin/matrigel-coated dishes (Figure 1).

**Figure 2:**
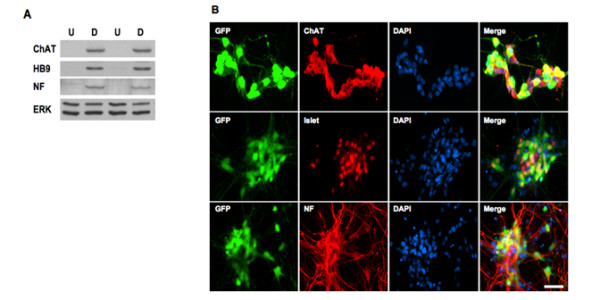
**Characterization of motor neuron differentiation**. HBG3 cells were induced to differentiate following the procedure as in 1A. Lysates from undifferentiated and differentiated cells were resolved on an SDS-PAGE and transferred to a membrane. The blot was probed with antibodies against pan-neuronal (neurofilament, NF) and motor neuron markers (Hb9 and ChAT). The same blot was stripped and re-probed with anti-ERK antibodies for protein loading control (A). Differentiated ES cells were dissociated and processed for indirect immunofluorescence staining for neurofilament (NF, in red), ChAT (in red), Islet-1 (in red), and DAPI (for nuclei in blue). Scale bar = 50 μm. U = undifferentiated cells and D = differentiated cells.

The differentiated cells were characterized by the expression of neuronal and motor neuron marker proteins. Western blotting shows that a pan-neuronal marker (neurofilament) and two motor neuron specific markers (Hb9 and ChAT) are expressed in differentiated ES cells but not in the undifferentiated ES cells (Figure 2A). Immunofluorescence staining shows that differentiated motor neurons are neurofilament, ChAT, and Islet-1 positive (Figure 2B).

### Proteomic analyses reveal altered protein expression in SMN-deficient ES cells

**Figure 3:**
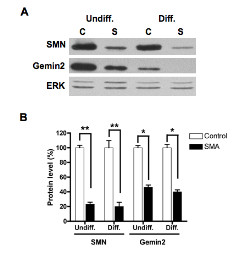
**Reduced expressions of SMN and Gemin2 in SMN-deficient ES cells**. Control (C) and SMA (S) ES cells were separated from feeding fibroblasts and allowed to differentiate *in vitro *in the presence of RA and SAG for 5 days. Both undifferentiated and differentiated cells were harvested and lysates were prepared. Equal amounts of total proteins were separated by SDS-PAGE, and SMN and Gemin2 were detected by western blotting. The same blot was stripped and re-probed with ERK antibodies for protein loading control (A). Signals were quantified, relative ratios of SMN or Gemin2 to ERK were calculated, and the mean value ± SD of three independent experiments is presented here. Statistical analyses (Student's *t *test) indicate that SMA cells have significantly reduced levels of SMN and Gemin2 expression (approximately 20% of the control for SMN and 40-50% for Gemin2) (** *p *< 0.01 and * *p *< 0.05) (B). Undiff. = undifferentiated cells and Diff. = differentiated cells.

**Figure 4:**
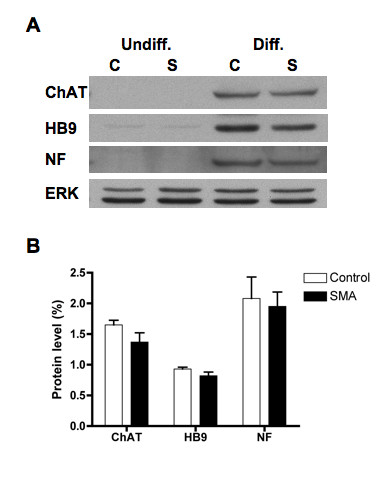
**Characterization of differentiation of control and SMN-deficient ES cells**. Control (C) and SMA (S) ES cells as described in 3A were processed for western blotting using antibodies against neurofilament (NF), Hb9, and ChAT respectively. The same blot was stripped and re-probed with anti-ERK antibody for protein loading control (A). Signals of three independent experiments were quantified, normalized to ERK, and plotted as for 3B. Differentiated control and SMA ES cells show similar levels of expression for neuronal and motor neuron marker proteins (B). Undiff. = undifferentiated cells and Diff. = differentiated cells.

The SMA ES cells were derived from a severe SMA transgenic mouse that lacks the murine *Smn *gene but carries two copies of the human *SMN2 *gene [25]. Western blotting shows that SMA ES cells have a marked reduction in SMN protein (~20% of control, Figure 3). The SMN-interacting protein Gemin2 was also significantly decreased (~40-50% of control, Figure 3). However, morphologically, SMA ES cells did not differ from the control ES cells (data not shown). Under the optimized differentiation conditions, SMN-deficient ES cells were differentiated into motor neurons *in vitro *as evidenced by neurofilament, ChAT, and Hb9 expression (Figure 4A). Despite a drastic difference in SMN levels (Figure 3), control and SMA ES cells showed similar levels of neuronal and motor neuron marker expression after differentiation (Figure 4B). This is consistent with immunostaining results showing that 45-50% of differentiated cells were Hb9^+^ in both control and SMA ES cells.

**Figure 5:**
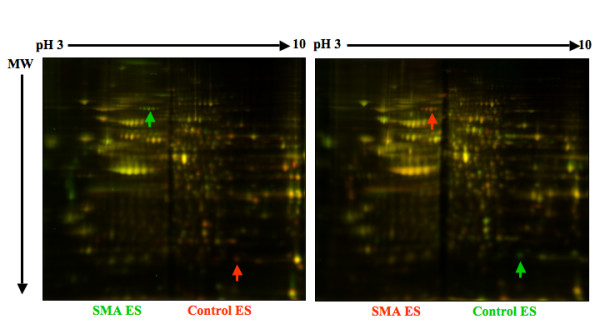
**Comparative 2D DIGE analysis of protein expression in control and SMA ES cells**. Control and SMA ES cells were harvested and lysed. Equal amounts of total proteins from wild-type or SMA ES cells were trace labeled with either Cy3 or Cy5. The dyed proteins indicated by the labeled colors were mixed and separated by isoelectric focusing in 7 cm pH 3-10 linear gradient IPG strips, and further separated in a second dimension of 10% SDS-PAGE. Fluorescently labeled proteins were visualized by Typhoon Trio scanner and difference between samples analyzed by the accompanied software. Samples shown in the left and right panels were from different preparations of ES cells and labeled with opposite dyes to demonstrate the reproducibility of the assay. A few abnormally expressed corresponding proteins were marked by arrows. MW = molecular weight.

**Figure 6:**
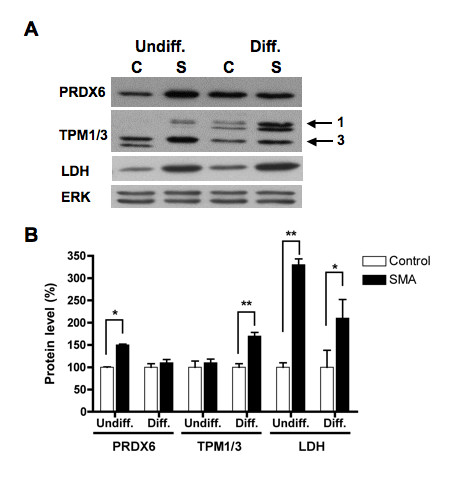
**Abnormal expression of peroxiredoxin 6, tropomyosin, and lactate dehydrogenase in SMN-deficient ES cells**. Control (C) and SMA (S) ES cells as described in 3A were processed for western blotting using antibodies against peroxiredoxin 6 (PRDX6), tropomyosin (TPM1/3), and lactate dehydrogenase (LDH), respectively. The same blot was stripped and re-probed with anti-ERK antibody for protein loading control (A). Signals of three independent experiments were quantified, normalized to ERK, and plotted as for 3B. Western blotting confirms that SMA ES cells show significantly higher levels of peroxiredoxin 6, tropomyosin, and lactate dehydrogenase expression (** *p *< 0.01 and * *p *< 0.05) (B). Undiff. = undifferentiated cells and Diff. = differentiated cells.

**Figure 7:**
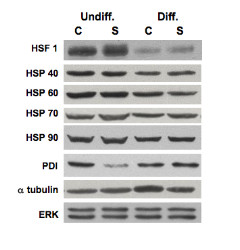
**Western blotting profile of α-tubulin and chaperone-related proteins**. Control (C) and SMA (S) ES cells as described in 3A were processed for western blot analyses using antibodies against α-tubulin, chaperone-related proteins as indicated, and protein loading control ERK. Undiff. = undifferentiated cells and Diff. = differentiated cells.

To identify proteins that are up- or down-regulated in SMN-deficient ES cells, comparative two-dimensional SDS-polyacrylamide gel electrophoresis (SDS-PAGE) analyses of control and SMA ES cells were performed. The differences between control and SMN-depleted protein pools were analyzed using four separate control and SMA ES cell extracts and quantified using DeCyder software (Figure 5). A total of 1900 spots were identified. Spots that differed with a p-value less than 0.05 (*t-*test) and that showed an average ratio of expression greater than or less than 1.5 were marked as points of interest (POI). For the undifferentiated ES cell samples, the 11 most abundant of the total 34 POIs were picked, and for the differentiated ES cell samples, the 14 most abundant of the total 32 POIs were picked. The protein identities of the picked POIs were determined by trypsin digestion and mass spectrometry. The majority of detected proteins were expressed at similar levels in control and SMA ES cells (Figure 5), but in our proteomic analyses, we were able to identify 4 out of 11 proteins in the undifferentiated samples and 11 out of 14 proteins in the differentiated samples that were significantly altered in SMA ES cells (Table 1). Most of the proteins identified as differentially expressed in SMA ES cells are involved in stress-responses (peroxiredoxin, stress induced phophoprotein, and chaperone proteins), cell metabolism (lactate dehydrogenase, glyoxalase 1, brain creatine kinase, and aldehyde dehydrogenase), protein turnover and ubiquitin modification (ubiquitin C-terminal hydrolase L1), and cytoskeleton stability (α-tubulin and tropomyosin 3). Consistent with our finding, ubiquitin C-terminal hydrolase L1 is up-regulated in type I SMA fibroblasts [41], and tropomyosin 3 is up-regulated while peroxiredoxin is down-regulated in the hippocampus of *Smn-/-; SMN2+/+ *mice [42]. We obtained antibodies to six of the differentially expressed proteins to validate the proteomic results. Western blot analysis confirmed that expression of peroxiredoxin 6 is increased in undifferentiated SMA cells and that lactate dehydrogenase and tropomyosin 3 are increased in differentiated SMA ES cells (Figure 6). However, no significant changes in levels of heat shock proteins 70/90 or tubulin were detected by western blotting (Figure 7). Western blotting showed that expression of lactate dehydrogenase was increased in both undifferentiated and differentiated SMA ES cells, indicating increased metabolic activity in SMN-deficient cells.

**Table 1:** Proteomic identification of dysregulated proteins in SMN-deficient ES cells

Protein Name	Gene Symbol	Fold Change	Function
			
**Undifferentiated**			
Chaperonin 6a	*Cct6a*	+2.6	Protein folding
FK506 binding protein 4	*Fkbp4*	+1.75	Protein folding and trafficking
Peroxiredoxin 6	*Prdx1*	+1.6	Redox regulation
Stress-induced-phosphoprotein 1	*Stip1*	-1.75	Adaptor protein for Hsp70 and Hsp90
			
**Differentiated**			
Lactate dehydrogenase B	*Ldhb*	+3.6	Glycolysis
Brain creatine kinase	*Ckb*	+1.8	Cellular energy homeostasis
Glyoxalase 1	*Glo1*	+1.75	Detoxification of glucose degradation products
Tropomyosin 3	*Tpm3*	+1.75	Stabilization of actin filaments
Annexin A5	*Anxa5*	+1.7	Cell signaling, Inflammation; Growth/differentiation
Ubiquitin carboxy-terminal hydrolase L1	*Uchl1*	+1.7	De-ubiquitination; Ubiquitin ligation; Mono-ubiquitin stabilizer
Alpha-tubulin	*Tuba1a*	+1.5	Cytoskeletal protein
Aldehyde dehydrogenase	*Aldh5a1*	-1.7	Aldehyde oxidation
14-3-3 gamma	*Ywhag*	-1.7	Cell signaling; Check point control; Apoptosis
Heat shock protein 90B	*Hsp90b1*	-1.8	Chaperone protein
Heat shock protein 70K	*Hspa9*	-2.2	Chaperone protein; Cell proliferation

### Western blot analysis reveals specific cell stress-response pathways activated in SMN-deficient ES cells

**Figure 8:**
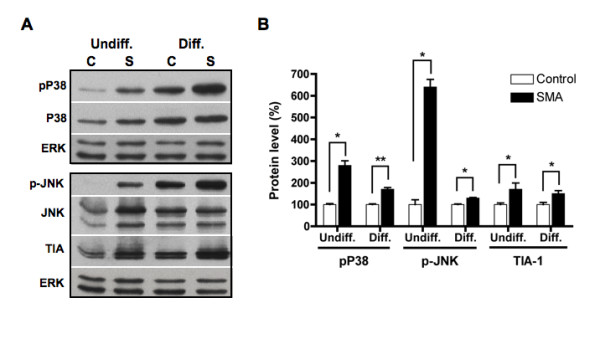
**Elevated expressions of stress-related proteins in SMN-deficient ES cells**. Control (C) and SMA (S) ES cells as described in 3A were processed for western blotting using antibodies against phosphorylated p38 (pP38), p38, phosphorylated JNK (p-JNK), JNK, and TIA-1. The same membranes were stripped and re-probed with anti-ERK antibody for protein loading control (A). Signals of three independent experiments were quantified, normalized to corresponding ERK signals, and plotted as for 3B. SMA ES cells express significantly higher levels of phosphorylated p38, phosphorylated JNK, and TIA-1 proteins (** *p *< 0.01 and * *p *< 0.05) (B). Undiff. = undifferentiated cells and Diff. = differentiated cells.

**Figure 9:**
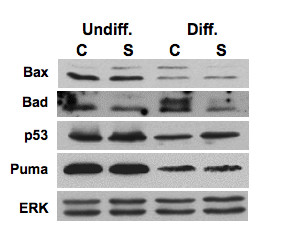
**Western blotting profile of cell death-related proteins**. Control (C) and SMA (S) ES cells as described in 3A were processed for western blot analyses using antibodies against Bax, Bad, Puma, p53, and protein loading control ERK. Undiff. = undifferentiated cells and Diff. = differentiated cells.

Our proteomic study indicates that reduced levels of SMN increase expression of stress-response proteins (Table 1). Specific stress-response pathways dysregulated in SMN-deficient ES cells were analyzed by western blotting. We first analyzed levels of the stress-activated protein kinase/Jun-amino-terminal kinase SAPK/JNK and p38 MAP kinase in SMN-deficient ES cells. JNK and p38 MAP kinase are the two major stress-response pathways that are activated by a variety of cellular stresses including UV light and growth factors [43,44]. Levels of phosphorylated p38 were elevated in SMA ES cells (~2.8 fold increase for the undifferentiated cells and ~1.7-fold increase for differentiated cells; Figure 8). Levels of phosphorylated JNK were also increased in both undifferentiated (~6.4 fold) and differentiated (~1.3 fold) SMA ES cells when compared to control ES cells. Up-regulation of phosphorylated JNK was previously seen in primary muscle cultures derived from SMA patients, although total levels of this protein were reduced in these cells [45]. Another cell stress-response protein that is often found in stress granules where SMN is seen to localize [46,47], TIA-1, was also up-regulated in SMA ES cells (~2 fold for undifferentiated cells and ~1.5 folds for differentiated cells). Activated in response to FAS ligand stimulation, TIA-1 appears to function as a mediator of apoptotic cell death [48]. Protein disulfide isomerase was down-regulated in SMA ES cells, but the vast majority of chaperones and ER stress-related proteins were not changed (Figure 7). Levels of p53, Bax, Bad, and Puma were also not affected in SMA ES cells (Figure 9).

### Cellular stress-response protein p21 is up-regulated in both SMN-deficient ES cells and in spinal cords of SMA transgenic mice

**Figure 10:**
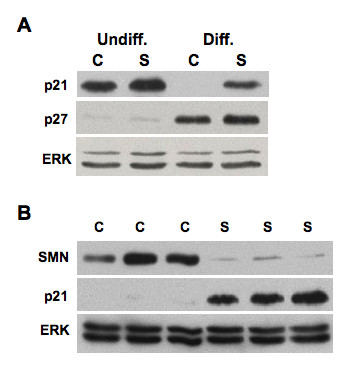
**Up-regulation of p21 in SMN-deficient ES cells and spinal cord tissues derived from SMA mice**. Control (C) and SMA (S) ES cells as described in 3A were processed for western blotting using antibodies against p21 and p27. The same membrane was stripped and re-probed with anti-ERK antibody for protein loading control (A). Spinal cord tissues from three control (C) and three SMA (S) mice were collected and lysates were prepared. Equal amount of protein lysates were separated on a SDS-PAGE and transferred to a membrane. The blot was probed with antibodies against p21 or SMN. The same blot was stripped and re-probed with anti-ERK antibody for protein loading control (B). Undiff. = undifferentiated cells and Diff. = differentiated cells.

The tumor suppressor protein p21 Waf1/Cip1 acts as an inhibitor of cell-cycle progression. It has been shown that p21 is induced in response to a variety of stress signals, including genotoxins, oxidants, and metabolic perturbation [49]. Western blot analyses show that levels of p21 are increased in both undifferentiated and differentiated SMA ES cells (Figure 10A). In differentiated control cells, p21 is expressed at very low levels but is increased by as much as 41.7 ± 0.3 fold (n = 4 independent experiments, *p *< 0.05, Student's *t-*test) in differentiated SMA cells. By contrast, the expression of another member of the Cip/Kip family of cyclin-dependent kinase inhibitors, p27, was unaffected (Figure 10A). p21 is also up-regulated in spinal tissue obtained from SMA transgenic mice compared to spinal tissue from control mice (17.1 ± 0.2 fold, *p *< 0.01, Student's *t*-test, Figure 10B).

## Discussion

In this study, we have developed an improved protocol for *in vitro *motor neuron differentiation using GFP expression as a readout in the control ES line (HBG3, HB9::eGFP) [31]. This protocol employs a multi-step process that involves priming ES cells with Noggin and FGFs prior to addition of signaling molecules RA and SAG (Figure 1). Using this modified procedure, we were able to generate a motor neuron population *in vitro *with high efficiency (~50%). The differentiated ES cells acquired immunohistochemical features of motor neurons as evidenced by expression of neuronal and motor neuron markers (Figure 2). We also derived ES cells from a severe transgenic SMA mouse for differentiation to provide a cell-culture model for type I SMA. Applying this method to SMA ES cells, we were able to perform proteomic analyses to identify pathways affected in both undifferentiated and differentiated SMA ES cells.

Differentiation efficiency of control and SMA ES cells were similar, but our study shows that several pathways involved in cell stress-response, energy metabolism, protein turnover, and cytoskeleton stability are affected by low expression of SMN (Table 1). Interestingly, similar disturbances in these pathways have been reported in other proteomic studies using different proteomic approaches that were conducted in SMN-deficient cells and tissues other than motor neurons [41,42,45,50,51].

Although motor neurons are the primary cells affected in SMA, most studies use alternative tissues or cell cultures because of difficulties in obtaining motor neurons from affected animals. Antibodies detect proteins with higher specificity and sensitivity, but such studies are restricted by availability of suitable antibodies for blotting or antibody arrays. By comparison, comparative two-dimensional SDS-PAGE difference gel electrophoresis and iTRAQ analyses can be used to provide unbiased analysis of protein changes. For iTRAQ expression, differences are determined from alterations in quantities of the tags used to label discrete populations of peptides derived from proteins isolated from control and affected cells or tissues. This requires all peptides to be identified by LC/MS/MS. For gel systems, differences in quantities of dye-tagged proteins are determined, and only differentially expressed proteins need to be processed for identification by MS/MS. We detected 1900 spots using two-dimensional SDS-PAGE compared to an iTRAQ study with SMA fibroblasts that detected 2171 proteins [50]. In both techniques, quantification of less-abundant proteins is less accurate. Among dysregulated proteins that are involved in cellular metabolism, we confirmed up-regulation of lactate dehydrogenase in SMA ES cells by western blotting (Figure 6). Lactate dehydrogenase is an oxidoreductase of the glycolysis pathway that catalyses the interconversion of pyruvate and lactate with concomitant interconversion of NADH and NAD+ [52]. Aberrant expression of lactate dehydrogenase and other energy metabolism enzymes (e.g., glyoxalase 1, brain creatine kinase, aldehyde dehydrogenase) in SMA cells could disturb energy production and consumption, and contribute to the pathology of SMA. Consistent with our findings, mRNAs encoding several enzymes of glycolysis are aberrantly expressed in muscles derived from SMA patients [53].

In our proteomic analyses, we also found up-regulation of tropomyosin in differentiated SMA ES cells (Table 1, Figure 6). A similar finding was reported in the hippocampus of *Smn-/-; SMN2+/+ *mice [42]. Tropomyosin is a dimeric coiled-coil protein that binds along the length of actin filaments. In non-muscle cells, tropomyosin stabilizes cytoskeleton actin filaments [54]. Thus, dysregulation of tropomyosin could compromise actin dynamics and cytoskeleton stability. Levels of other actin binding proteins have been proposed to play a role in SMA. Profilin II, the small actin-binding protein that associates with SMN [55,56], is up-regulated in both SMN-depleted PC12 cells and cells from an SMA mouse model [57,58]. The actin bundling protein Plastin 3 has recently been identified as a protective, gender-specific modifier of SMA and is associated with SMN and actin in a large protein complex [59]. Together, these findings implicate a role for disruption of actin cytoskeletal dynamics in SMA pathogenesis.

Up-regulation of peroxiredoxin 6 in SMA ES cells (Table 1 and Figure 6), and increased expression of peroxiredoxin protein in SMA mouse tissue [42], implicates cell stress-responses in SMA. Peroxiredoxin 6 is an antioxidant enzyme that reduces levels of H_2_O_2 _to protect cells from oxidative injury [60]. Up-regulation of peroxiredoxin 6 could be a compensatory response to potentially increased oxidative stress in SMN-deficient cells. Reduced levels of SMN have been shown to cause mitochondrial dysfunction [61], which can initiate oxidative stress. Expression of peroxiredoxin 6 is also increased in a mutant SOD1 mouse model of amyotrophic lateral sclerosis [62]. Interestingly, over-expression of SMN protects cells against cell death induced by mutant SOD1 under oxidative stress [63]. To investigate the notion that SMN deficiency might lead cells in stress in more detail, we used western blotting to show that activation of the p38 MAP kinase and JNK pathways were increased in both undifferentiated and differentiated SMA ES cells (Figure 8). Both of these pathways have been implicated in neuronal cell death by activating apoptotic pathways in response to heat and osmotic shock, genotoxic damage, X-ray and UV radiation, and proinflammatory cytokines [64,65]. We also showed that the stress-response protein TIA-1 is increased in SMA ES cells (Figure 8). TIA-1 is often localized in stress granules where SMN is seen to locate [47]. Notably, expression of the stress-response protein p21 is increased significantly in both SMA ES cells and spinal cord tissues of a mouse model of SMA (Figure 10). We could not determine whether disease progression affects p21 expression because we were unable to recover sufficient spinal cord samples from different aged SMA mice. Although p21 expression is higher in undifferentiated SMA cells than in undifferentiated control cells, the differences are much more striking in the differentiated cells. Up-regulation of p21 is a cellular response to stress that leads to cell growth arrest and favors cell survival [66], but prolonged stress in SMA cells will eventually lead to cell death. Transcription factors such as p53, Sp1, p300, CEBPβ, and Stats induce expression of the p21 gene [49,67]. However, as p53 levels are not altered in SMN-deficient cells (Figure 9), it is unlikely that up-regulation of p21 is mediated by this transcription factor in SMN-deficient cells. Thus, the mechanism by which SMN deficiency leads to up-regulation of p21 is not yet clear.

Our analyses show a number of pathways that are dysregulated in SMA ES cells but the most important may be induction of cell stress. This cell stress might explain why we see significantly reduced numbers of SMA ES cells compared to control ES cells 5 days after differentiation (C. Wu, D. Whye, W. Wang, unpublished data) and why motor neurons are particularly sensitive to SMN deficiency in SMA patients. Stress-induced cell death is more pronounced in fibroblasts derived from SMA patients compared to age-matched controls [68], and regulation of SMN expression directly influences cell survival [69-72]. Modulation of levels of down-stream effectors of cell death such as pro-apoptotic Bax and the antiapoptotic factor Bcl-x_L _in SMA mice directly impacts the disease phenotype and lifespan of SMA mice [73,74]. All these studies support the hypothesis that SMN plays an active role in cell survival. The recent groundbreaking discovery that human fibroblasts can be reprogrammed into an ES cell-like state has significantly advanced the field of stem cell and neurodegenerative disease research. Therefore, it will be of great interest to determine if the similar pathways are aberrantly regulated in the induced pluripotent stem cells derived from SMA fibroblasts.

## Conclusion

We have derived SMN-deficient ES cells from a severe transgenic SMA mouse which represent a cell-culture model of SMA. Our work indicates that lower expression of SMN activates cellular stress pathways and causes a dysregulation of energy metabolism, protein degradation, and cytoskeleton stability.

## Methods

### Embryonic stem cell culture

HBG3 ES cells were derived from a HB9::GFP (*mHB9-Gfp1b*) transgenic mouse as previously reported [31], and control (*SMN2+/+; Smn+/+) *and SMA (*SMN2+/+; Smn-/-*) murine ES cells were derived from a SMA transgenic mouse (*FVB.Cg-Tg(SMN2)89Ahmb Smn1*^*tm1Msd*^*/J*) [25] using similar techniques. All mice were obtained from Jackson Laboratories, Bar Harbor, ME, USA. All procedures involving animals for deriving ES cells were performed in conformity with the guidelines of the National Institutes of Health and were approved by the Johns-Hopkins University Institutional Animal Care and Use Committee (approved protocol number: MO06M240). Embryonic stem cells were expanded on feeder layers of primary mouse embryonic fibroblasts (PMEF, hygro-resistant, passage 3, PMEF-H; Millipore, Billerica, MA, USA) in 100-mm tissue culture dishes. Cells were cultured with ES cells growth medium containing DMEM supplemented with 15% fetal bovine serum (FBS, Stem Cell Technologies, Vancouver, BC, Canada), 1% GlutaMax-I supplement, 1% MEM non-essential amino acids, 1% nucleosides, 0.1 mM β-mercaptoethanol, 1% penicillin/streptomycin, and 1 μl/ml murine leukemia inhibitor factor (Millipore). Whenever possible, stem-cell grade reagents were used for ES cell culture.

### Differentiation of ES cells into motor neurons

Embryonic stem cells on fibroblast feeder layers were dissociated with 0.25% trypsin/EDTA and plated onto 0.1% gelatin-coated tissue culture flasks. Fibroblasts were allowed to re-attach to the flask, and then the floating ES cell population was collected. Approximately 2 × 10^6 ^cells were plated into 100-mm petri dishes containing 10 ml of neural differentiation priming medium containing DMEM supplemented with 15% FBS, 1% MEM non-essential amino acids, 1% GlutaMax-I supplement, 1% penicillin/streptomycin, 1 mM monothio-glycerol, 50 ng/ml Noggin (Invitrogen, Carlsbad, CA, USA), 20 ng/ml bFGF (Invitrogen), and 20 ng/ml FGF-8 (Invitrogen) for EB formation. Media were replaced daily. After two days, aggregated EBs were re-suspended in motor neuron differentiation medium (NITSf) containing basal medium A supplemented with 10% knockout serum replacement (Invitrogen), 1% N-2 supplement, 1% ITS-B supplement (Stem Cell Technologies), 1% ascorbic acid, 1% penicillin/streptomycin, 0.1 mM β-mercaptoethanol, 0.5% GlutaMax-I supplement, 30% D-glucose, 20 μg/ml heparin (Sigma, St. Louis, MO, USA) and 50 μg/ml fibronectin (Stem Cell Technologies) in the presence of 1 μM RA (Sigma) and 1 μM SAG (Calbiochem, Gibbstown, NJ, USA). Media were replaced daily. After 5 days of induction with RA and SAG, EBs were collected, washed with phosphate-buffered saline (PBS), and dissociated in Accumax (Millipore) following the manufacturer's protocol. Cell aggregates were depleted from dissociated EBs by filtering through a BD cell strainer. Single cells were then suspended in NITSf medium supplemented with 10 ng/ml of each BDNF, GDNF, CNTF, and NT-3 and plated at a concentration of 1 × 10^5 ^cells per well of 24 well plates that contained poly-DL-ornithine hydrobromide (Sigma)/laminin (Millipore)/matrigel- (BD Bioscience, San Jose, CA, USA) coated coverslips.

### Comparative two-dimensional SDS-polyacrylamide gel electrophoresis and mass spectrometry analysis

Undifferentiated and differentiated control and SMA ES cells were lysed in 30 mM Tris, pH 8.5 containing 7 M urea, 2 M thiourea, and 4% CHAPS. Lysates were cleared by centrifuging at 12,000 × g at 4ºC for 10 min. A portion of each sample supernatant was pooled together to create an internal standard. Individual samples were labeled with CyDye DIGE Fluor minimal labeling dyes (GE Healthcare, Piscataway, NJ, USA) at a ratio of 200 pmol Cydye per 50 μg protein according to manufacturer's protocols. For each experiment, two control and two SMA samples were labeled with Cy3, and another two control and two SMA samples were labeled with Cy5. This dye swap was performed to negate any dye preference artifacts in analysis. The internal standard protein pool was labeled with Cy2. Equal portions of control, SMA, and internal standard were mixed and loaded onto IPG strips (BioRad, Hercules, CA, USA) via rehydration in the presence of the same lysis buffer (see above) containing 0.5% Pharmalyte 3-10 (BioRad). Samples from undifferentiated ES cells were run on pH 3-10 linear IPG strips, while differentiated samples were run on pH 3-10 non-linear IPG strips. After isoelectric focusing, IPG strips were equilibrated for 15 min with 75 mM Tris, pH 8.8, 6 M Urea, 30% Glycerol, 2% SDS, and 65 mM DTT followed by 15 min with 75 mM Tris, pH 8.8, 6 M Urea, 30% Glycerol, 2% SDS, and 135 mM iodoacetamide (Sigma). The IPG strips were loaded onto 10% SDS-PAGE gels, and proteins were separated by electrophoresis and imaged using a Typhoon Trio Scanner (GE Healthcare). Changes in levels of individual protein spots were determined using Decyder 6.5 software (GE Healthcare). Statistical significance of the change was determined using a paired, two-tailed Student's *t*-test. Spots were ranked according to p-values. Spots with a *t*-test p-value of less than 0.05 and an average ratio greater than or less than 1.5 were picked and trypsin digested.

Peptide identities were determined by mass spectrometry. Matrix-assisted laser desorption ionization time-of-flight mass spectrometry (MALDI TOF MS) was carried out on a 4800 MALDI TOF/TOF Analyzer (Applied Biosystems, Atlanta, GA, USA). Peptide mass fingerprint (PMF) spectra were obtained in positive ion reflector mode with internal calibration for each sample. Four peaks from each spectrum were selected and subjected to further fragmentation for sequence information via tandem MS (MS/MS) with default calibration. Using the search program Mascot (v2.2, Matrix Sciences), PMF and MS/MS spectra were submitted for database searches with the following parameters: NCBInr database, trypsin enzyme, 1 maximum missed cleavage, oxidation of methionines, carbamidomethylation of cysteines, 75 ppm precursor mass tolerance, and 0.3 Da MS/MS fragment mass tolerance. Only protein matches with a probability score below the significance threshold (*p *< 0.05) were considered.

### Immunocytochemistry and western blot analysis

Immunocytochemistry and western blot analyses were performed as previously described [68,75]. In brief, differentiated ES cells were washed in PBS, fixed with 4% paraformaldehyde in PBS for 10 min, and permeabilized in 0.1% NP-40 in PBS for 15 min. After two washes with 1× PBS, cells were blocked in 5% BSA in Tris-buffered saline containing 0.05% Tween-20 (TBS-T) for 1 hr at room temperature and then incubated overnight at 4°C with mouse anti-Islet-1 (1:50, 39.4D5, Developmental Studies Hybridoma Bank, Iowa City, IA, USA), goat anti-choline acetyltransferase (1:200, Millipore), or chicken anti-neurofilament (1:200, Millipore). After washing three times with TBS-T, the cells were incubated with donkey anti-mouse Texas Red, donkey anti-chicken Texas Red, or donkey anti-goat DyLight 549. All secondary antibodies were purchased from Jackson ImmunoResearch (West Grove, PA, USA). Slides were mounted in Vectashield containing DAPI (Vector Laboratories, Burlingame, CA, USA).

For western blotting, spinal cord samples were collected at P2-5 from the same colonies of transgenic mice used to derive control and SMA ES cells (see above). All procedures involving animals for collecting spinal cord tissues were performed in conformity with the guidelines of the National Institutes of Health and were approved by the Nemours/Alfred I. duPont Hospital for Children Institutional Animal Care and Use Committee (approved protocol number: NBR-2008-003). Prior to tissue harvesting, the genotype of SMA pups were determined by standard PCR (Jackson laboratories). Lysates from spinal cord tissues and ES cells were prepared, protein concentrations were measured using the BCA assay, and western blot analyses were performed as previously described [68,75]. In brief, 50 μg of total protein lysates were resolved by 10% SDS-PAGE and transferred to nitrocellulose membranes. The membranes were probed with the following antibodies: neurofilament (1:500, Millipore), ChAT (1:500, Millipore), Hb9 (1:10,000, Abcam, Cambridge, MA, USA), SMN (1:1000, BD Biosciences), Gemin2 (2E17, 1:1000, Abcam), peroxiredoxin 6 (1:1000, Abcam), lactate dehydrogenase (1:5000, Abcam), tropomyosin 1/3 (1:1000, Cell Signaling, Danvers, MA, USA), p38, p-p38, JNK, p-JNK (1:1000, Cell Signaling), p21 (1:500, Cell Signaling), p27 (1:2500, BD Bioscience), TIA-1 (1:500, Santa Cruz, Santa Cruz, CA, USA), and Erk (1:10,000, Santa Cruz). The blots were then incubated with the appropriate secondary HRP-conjugated antibodies, and proteins were detected using enhanced chemiluminescence (GE Healthcare). Signals were quantified, and ratios of proteins of interest to Erk were calculated as previously described [68,75]. Statistical analyses were determined using a paired, two-tailed Student's *t*-test (GraphPad Prism software, La Jolla, CA). P-values less than 0.05 were considered to be statistically significant.

## Competing interests

The authors declare that they have no competing interests.

## Authors' contributions

CW bred mice, collected and genotyped spinal cord samples, performed immunofluorescence studies of differentiated ES cells, and participated in writing the manuscript. DW developed the protocol for ES cell differentiation and participated in western blot analyses and manuscript writing. LG and RWM carried out proteomic work including 2D SDS-PAGE analyses, spot picking, and trypsin digestion. DK contributed ES cells. LC and KHL conducted mass spectrometry studies. WW participated in western blot analyses, overall design of the study, and the writing of the manuscript. All authors read and approved the final manuscript.
